# Electrocardiogram Heartbeat Classification for Arrhythmias and Myocardial Infarction

**DOI:** 10.3390/s23062993

**Published:** 2023-03-09

**Authors:** Bach-Tung Pham, Phuong Thi Le, Tzu-Chiang Tai, Yi-Chiung Hsu, Yung-Hui Li, Jia-Ching Wang

**Affiliations:** 1 Department of Computer Science and Information Engineering, National Central University, Taoyuan City 320317, Taiwan; 2 Department of Biomedical Sciences and Engineering, National Central University, Taoyuan City 320317, Taiwan; 3 Department of Computer Science and Information Engineering, Providence University, Taichung City 43301, Taiwan; 4 AI Research Center, Hon Hai Research Institute, New Taipei City 236, Taiwan

**Keywords:** electrocardiogram (ECG) classification, MIT-BIH dataset, PTB dataset, deep learning

## Abstract

An electrocardiogram (ECG) is a basic and quick test for evaluating cardiac disorders and is crucial for remote patient monitoring equipment. An accurate ECG signal classification is critical for real-time measurement, analysis, archiving, and transmission of clinical data. Numerous studies have focused on accurate heartbeat classification, and deep neural networks have been suggested for better accuracy and simplicity. We investigated a new model for ECG heartbeat classification and found that it surpasses state-of-the-art models, achieving remarkable accuracy scores of 98.5% on the Physionet MIT-BIH dataset and 98.28% on the PTB database. Furthermore, our model achieves an impressive F1-score of approximately 86.71%, outperforming other models, such as MINA, CRNN, and EXpertRF on the PhysioNet Challenge 2017 dataset.

## 1. Introduction

**Figure 1:**
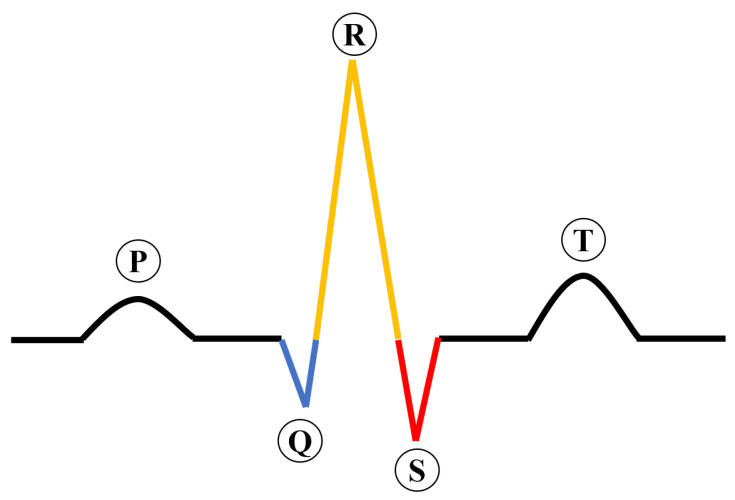
The construction of an ECG signal.

Cardiovascular diseases (CVDs) have surpassed cancer as the number one killer globally, killing approximately 17.3 million people yearly [1]. In the United States, CVDs accounted for 874,613 deaths in 2019, as reported by the American Heart Association. An ECG is a commonly used diagnostic tool in healthcare settings for assessing cardiovascular function [2,3]. Figure 1 describes the construction of an ECG signal, which is important for interpreting the results of the test.

An electrocardiogram (ECG) consists of five waves: P, Q, R, S, and T. The P wave indicates atrial contraction, and the T wave indicates ventricular repolarization. The QRS complex, which is a key component of an ECG, is composed of Q, R, and S waves. The QRS complex represents the electrical activity of ventricular depolarization as it spreads through the ventricles. The T wave, which follows the QRS complex, is an indicator of ventricular repolarization.

Electrocardiogram (ECG) monitoring shows the electrical activity of the heart, which is recorded as an electrocardiographic signal. This signal can be used to identify abnormal heart rhythms and other heart-related conditions, contributing significantly to the prediction of heart diseases. Wearable devices can monitor heart conditions based on the frequency of contractions during ECG measurements without requiring a consultation with a physician. Arrhythmia classification is another application of ECG monitoring. This measurement contributes significantly to the prediction of heart diseases [4,5,6,7], arrhythmia classification [8], cardiovascular disease [9], stroke disease [10], and the detection of atrial fibrillation [11]. Recently, the ECG has been one of the tools used for COVID-19 diagnosis [12,13,14,15]. As a result, the ECG monitoring system is used in hospitals [16,17], smart homes [18,19], remote contexts [20], or sports [21,22]. In reality, there are many available techniques, such as wearable and portable devices, which make embedding ECG systems into them convenient and appropriate for remote patient monitoring. This application not only reduces the risk of stroke or sudden death in patients but also lowers the risk of disease transmission. Moreover, patients can receive timely treatment and recover sooner. To achieve this goal, a low-cost ECG diagnosis system for arrhythmic heartbeats with high accuracy is highly desirable. Such a system reduces the workload of doctors and improves the efficiency of ECG diagnosis, as relying solely on labor-intensive readings by doctors has a high rate of misdiagnosis and a lengthy processing time. Many ECG datasets and research proposals have been recently published to recognize ECG signals; however, the time-series data of the signal with different waveforms and morphologies challenge the ECG data, leading to signal misinterpretation. Additionally, the signals are inevitably contaminated by noise sources, such as electrical interfaces. Three well-known datasets for ECG diagnosis are the PhysioNet MIT-BIH arrhythmia dataset [23], the PTB diagnostics dataset [24], and the PhysioNet Challenge 2017 dataset [25]. There are two main approaches to ECG diagnosis, namely classical and deep learning.

Some classical methods, such as the decision tree [26], were used for ECG heartbeat classification. ECG heartbeats can be classified using support vector machines (SVM) and the Naive Bayes Classification algorithm. Other algorithms for ECG heartbeat classification are multimodal feature fusion and multimodal image fusion (MIF) [27]. A decision tree also was proposed in [28] for analyzing ECG signals. Wavelet transform (WT), independent component analysis (ICA), and interval information (RR) are three algorithms for detecting and classifying ECG signals [29]. The discrete cosine transform (DCT) and Fisher’s linear discriminant analysis have been the approaches to analyzing features and classifying arrhythmias [30]. These algorithms extract features from the ECG signal, such as frequency, temporal, statistical, and so on. These features were then used to classify ECG heartbeats. Although these algorithms are basic and simple methods of ECG heartbeat classification, the results must improve. An F1-score of 0.87 was achieved by the SVM algorithm.

Some deep learning models apply to heartbeat classification, such as convolutional neural networks (CNN) [31,32], artificial neural networks (ANN) [33], long short-term memory (LSTM) [34,35]. To classify the ECG image types in a large dataset, previous studies have used ResNet, AlexNet, and SqueezeNet [36]. In general, deep learning models have been found to have superior performance compared to classical methods in classifying ECG signals. However, these models have been trained on smaller datasets of heartbeats and have a larger number of layers, which could be a reason for this high performance. Deep learning models are easily prone to over-fitting. In addition, a sizeable learning model is a big problem, resulting in long processing times, high computational resources, a large memory, and inefficient mobile solutions for long-term ECG monitoring. In this work, we introduce a new method for distinguishing electrocardiogram (ECG) signals. The novel approach suggested in this study includes the use of evolving normalization, residual block, gradient clipping, and a normalized gradient, which make it more generalizable and computationally efficient for classifying arrhythmias. The evolving normalization is fed into a residual block, resulting in a significant improvement in the performance of classifying various ECG signals. The effectiveness of our method is assessed using three publicly accessible ECG datasets. The results of the experiments demonstrate that the proposed model significantly enhances the classification of ECG heartbeats.

The primary advancements of this research can be summarized as follows:

- The examination of a selection of current neural network methods for the identification of electrocardiogram signals;
- The introduction of a new model for classifying electrocardiogram (ECG) signals;
- The examination of the efficiency of the proposed model on three published ECG datasets.

## 2. Related Work

Deep learning is a state-of-the-art method for extracting features, predicting, detecting, making decisions, and classifying different classes using a set of datasets. One of the major advantages of deep learning methods for ECG classification is that they can learn complex relationships between the ECG signal and various cardiovascular conditions. For instance, deep neural networks can automatically learn features of ECG signals, such as their shape, frequency, and amplitude, that indicate specific heart conditions, thereby improving upon traditional ECG classification methods. Another advantage of deep learning for ECG classification is its ability to handle large and noisy datasets, which are common in healthcare. Deep neural networks can effectively learn from ECG signals with various noises and artifacts and generalize well to unseen data. Due to their high efficiency, many studies have proposed using deep learning models for ECG classification. This study reviews three types of neural networks for ECG classification: convolutional neural networks (CNN), recurrent neural networks (RNN), and LTSM. However, interpreting the results from these deep learning methods depends on various factors, such as the hardware platform, the model’s architecture, and compiler optimization, which can directly impact training the model.

### 2.1. Convolutional Neural Network (CNN)

Convolutional neural networks have gained widespread use in the field of computer vision [37,38]. A CNN consists of three important layers: the input layer, multiple hidden layers, and the output layer. The hidden layers are made up of three layer categories: convolutional, pooling, and fully connected layers. The convolutional layers in a CNN identify features within the input data, while the pooling layers decrease the dimensions of the feature map. The fully connected layers classify these features. Acharya et al. [31] used 13 layers to classify electrocardiogram (ECG) signals into three categories: normal, preictal, and seizure, but their experiment used only a small dataset. Panda et al. [39] applied CNN to process ECG signals and showed that their method could only classify two classes. Other studies based on VGG-Net proposed a 2D CNN that can classify eight classes [40]. This study used optimization techniques, such as K-fold cross-validation, data augmentation, and regularization, to solve the overfitting problem and improve the classification’s performance. Anwar et al. [41] presented a framework with four steps: preprocessing, heartbeat segmentation, feature extraction, and heartbeat classification. They used discrete wavelet transform (DWT), RR interval, and the Teager energy operator (TEO) to extract features before the neural network classifier. Fatma et al. [42] suggested a deep-learning model for classifying five-class electrocardiogram (ECG) datasets. Ullah et al. [43] used a deep CNN with a pretrained ResNet-18 to identify premature ventricular contraction (PVC) on the MIT-BIH dataset and the Institute of Cardiological Technics (INCART), respectively. Naz et al. [44] proposed using a deep CNN with a pretrained AlexNet, VGG19, and Inception-v3 for the diagnosis of VTA ECG signals.

### 2.2. Recurrent Neural Network (RNN)

RNN is widely used in natural language processing [45]. Similar to convolutional neural networks (CNNs), the structure of RNNs includes an input layer, a series of hidden layers, and an output layer. However, the hidden layers in RNNs are composed of recurrent layers and fully connected layers, as opposed to convolutional and pooling layers in CNNs. RNNs utilize recurrent layers to identify temporal patterns in the input data by processing the input data in a sequential manner. The fully connected layers then categorize the features extracted from the recurrent layers. AI-Zaiti et al. [33] also introduced DNN to analyze ECG signals. An early deep learning approach method for ECG signal classification was found to be effective in the training dataset, as evidenced by its high performance metrics. However, when tested on an independent dataset, the model demonstrated poor generalizability, indicating that it may not perform well on unseen data. Xiong et al. [46] developed RhythmNet to recognize ECGs of different rhythms. RhythmNet can help distinguish between atrial fibrillation (AF) and normal rhythms. Additionally, the proposed model utilizes a combination of convolutional and recurrent layers to identify patterns and make predictions in the ECG signal. Specifically, the model includes sixteen convolutional layers with a filter size of 15 × 1, three recurrent layers, and two fully connected layers. This design allows for efficient feature extraction of the ECG signal. However, it is worth highlighting that the model demonstrated an efficient performance on the training dataset but had poor generalizability on the testing dataset. They applied softmax activation to calculate the probability of each class. In their experiment, the margin of error between normal rhythm and AF was limited, implying that RhythmNet was adept at distinguishing between these two categories.

### 2.3. Long Short-Term Memory (LSTM)

LSTM has shown promising results in various applications, including disease prediction and ECG signal classification [47]. Studies such as Yildirim et al. [34], Saadatnejad et al. [35], and Hou et al. [48] have applied LSTM in different ways to improve the efficiency and accuracy of ECG signal classification. Yildirim et al. [34] proposed the use of a new input layer, called the wavelet sequence (WS), which improved the efficiency of the LSTM network. Saadatnejad et al. [35] presented a lightweight model using wavelet transform and multiple LSTM layers, while Hou et al. [48] introduced a deep learning model with an encoder and a decoder, which used LSTM networks to extract high-level features and classify the signals into different categories.

Overall, the previous studies have shown that recent 2DCNN methods have promising classification performances. However, these methods require the transformation of ECG sequence data to the two-dimensional domain and are very computationally intensive, as seen in VGG, ResNet, and Inception-v3. On the other hand, RNN-LSTM has also demonstrated good performance, but the method focuses heavily on the temporal domain, which can lead to overfitting and poorer results on test data. In this work, we aim to improve the accuracy of ECG signal classification by developing a novel 1DCNN method that incorporates evolving normalization–activation (EVO), squeeze-and-excitation (SE), and gradient clipping (GC) components. Our approach outperforms existing techniques, achieving a significant improvement in classification accuracy for several datasets. By optimizing these components, we were able to effectively address the limitations of the previous 2DCNN and RNN-LSTM methods and achieve state-of-the-art results for ECG signal classification tasks.

## 3. Materials and Methods

### 3.1. Dataset

It is worth noting that the PhysioNet MIT-BIH Arrhythmia dataset [49] and the PTB Diagnostic ECG dataset [50] are widely used in the field of ECG signal analysis and have been used in many previous studies for algorithm development, testing, and comparison. These datasets are publicly available and free to download, making them an accessible resource for researchers and developers. Additionally, the annotations provided with these datasets are considered to be reliable and accurate, as they were determined by multiple experts in the field. However, it is important to note that the datasets have their limitations, including the small size of the MIT-BIH dataset and the limited scope of diagnoses in the PTB dataset. As such, caution should be taken when generalizing findings from these datasets to larger and more diverse populations.

The MIT-BIH Arrhythmia dataset is a set of ECG recordings obtained from 47 participants, comprising 48 two-channel, half-hour ambulatory recordings. The data were recorded at 360 samples per second per channel, using an 11 bit resolution and a 10 mV range. The dataset also includes reference annotations for each beat, which were determined by two or more cardiologists and any discrepancies were resolved. The dataset includes approximately 110,000 annotations in total, with the corresponding beat labels in the accompanying Table 1.

The PTB Diagnostics dataset is composed of ECG signals obtained from 290 individuals, including 148 diagnosed with myocardial infarction (MI) and 52 healthy controls, as well as individuals diagnosed with various other conditions. Each record includes ECG measurements taken from 12 different leads, all sampled at a frequency of 1000 Hz. In this particular study, only the ECG Lead II was utilized, and the focus was classification of the MI and healthy control groups.

For our study, we utilized ECG data from the PhysioNet Challenge 2017 dataset [25]. This dataset contains a total of 8528 ECG recordings sampled by the AliveCor device at 300 Hz, with durations ranging from 9 s to over 60 s. Of these recordings, 738 were from patients with AF, while the remaining 7790 were from non-AF candidates. The number of recordings for each group is summarized in Table 2. We chose this dataset because of its large size and the availability of annotated labels, which were determined by experts and verified by a second reader.

#### Pre-Processing Data

**Figure 2:**
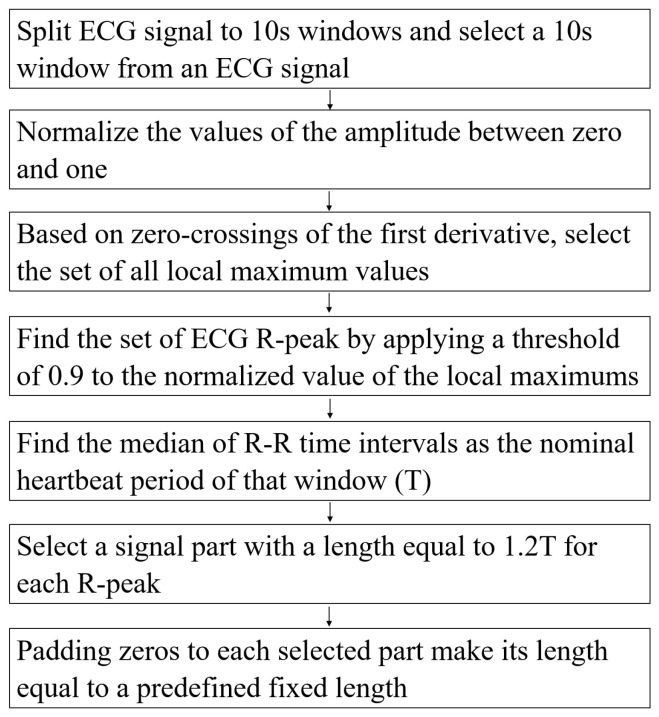
The pre-processing steps.

The authors in [51] detailed the pre-processing steps. In this study, we follow their steps to extract beats from an ECG signal. The pre-processing steps are described in Figure 2.

**Figure 3:**
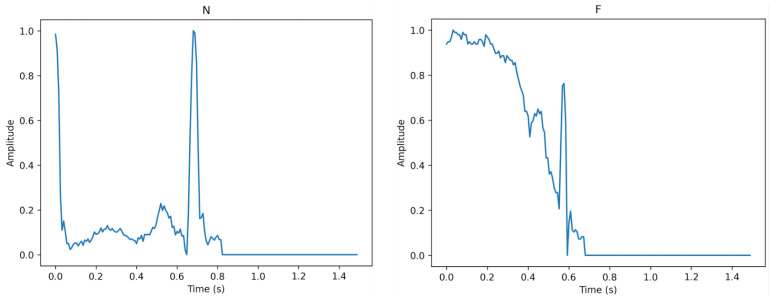
The description of class N (**left**) and class F (**right**).

**Figure 4:**
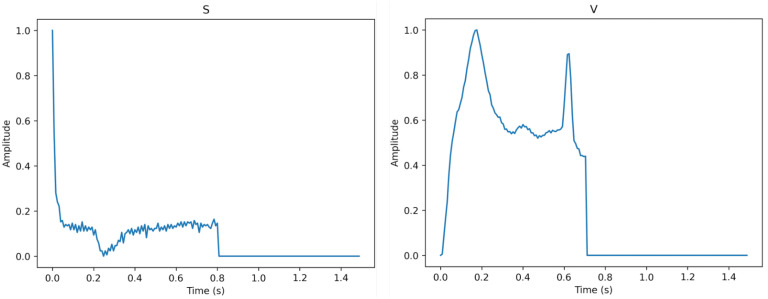
The description of class S (**left**) and class V (**right**).

**Figure 5:**
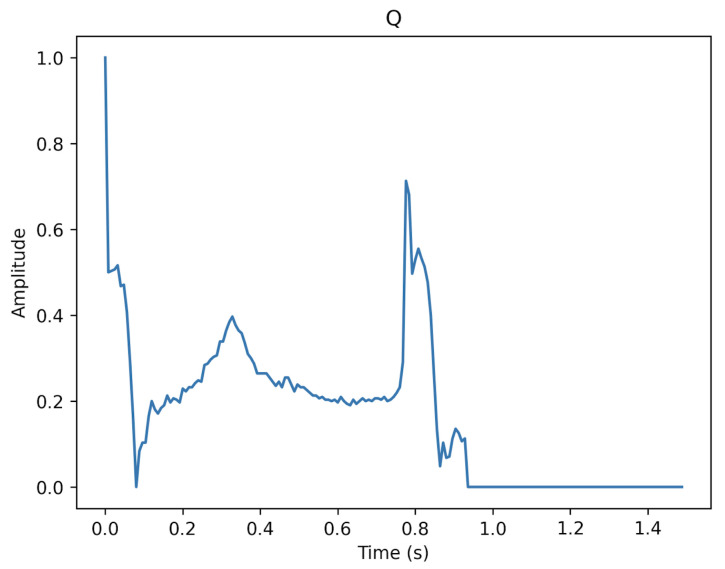
The description of class Q.

Figure 3, Figure 4 and Figure 5 show the specifics of each class. The variations in amplitude at different times represent the unique characteristics of each class.

**Figure 6:**
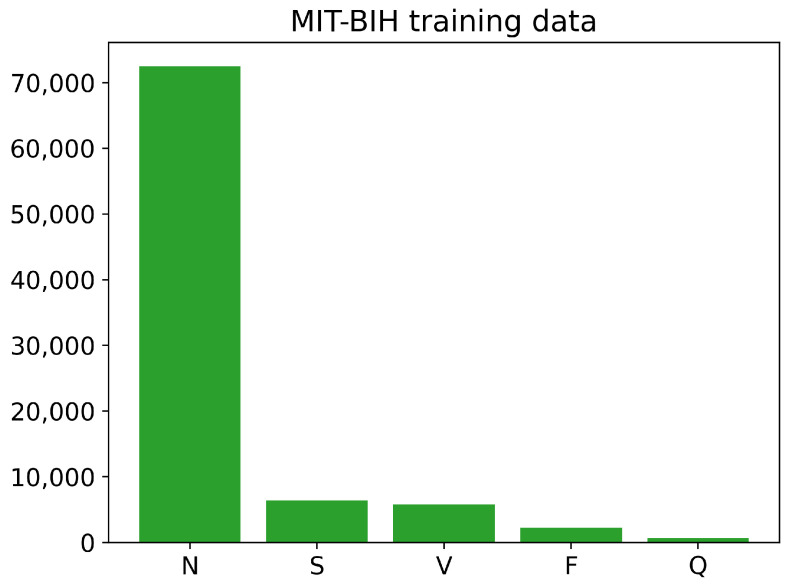
The number of images of each class in training PhysioNet MIT-BIH Arrhythmia data.

**Figure 7:**
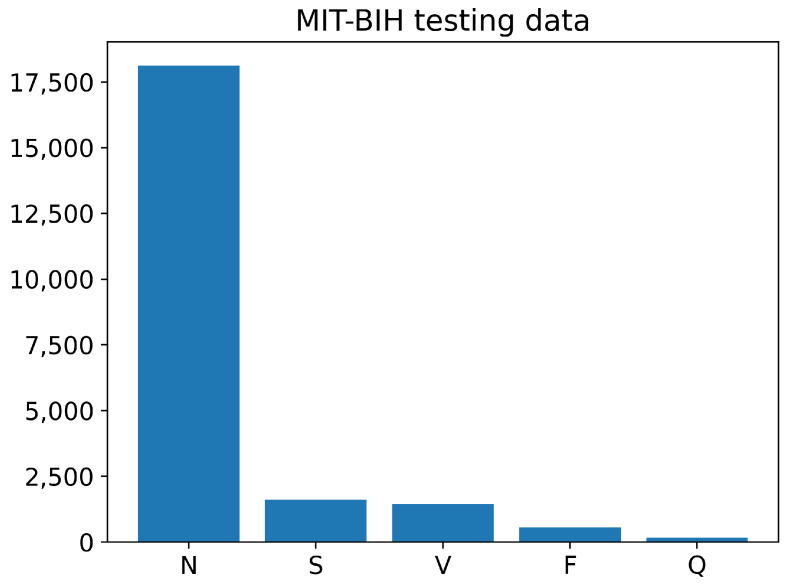
The number of images of each class in testing PhysioNet MIT-BIH Arrhythmia data.

The training and testing sets of the PhysioNet MIT-BIH Arrhythmia data contain different numbers of images for each class, as shown in Figure 6 and Figure 7. The training data have a large number of images belonging to class N, with over 70,000 images, while the number of training images for classes S, V, and F is less than 10,000. The Q class has the least number of training images. Similarly, the testing data also have an imbalanced distribution of images across the different classes. Overall, the training and testing sets of the PhysioNet MIT-BIH Arrhythmia data exhibit an imbalance in the number of images per class.

**Figure 8:**
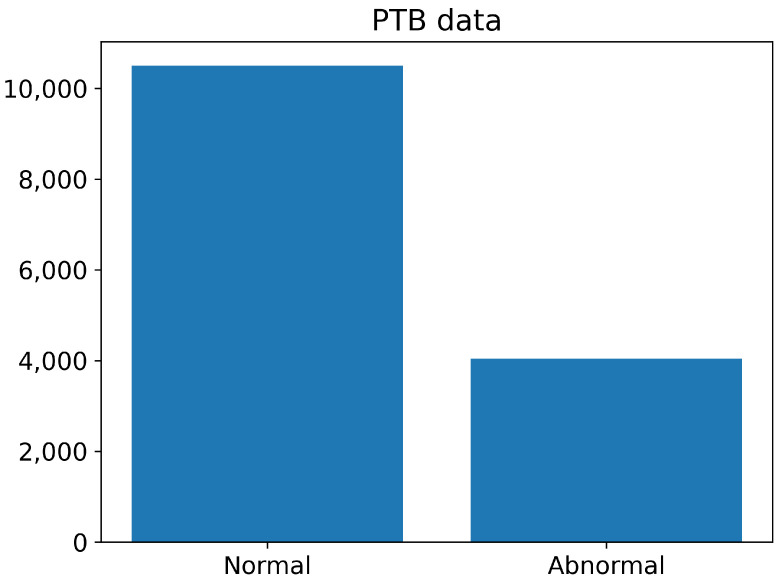
The number of normal and abnormal images of the PTB Diagnostics dataset.

The number of normal and abnormal images of the PTB Diagnostics dataset are summarized in Figure 8. The number of normal images is greater than 10,000, whereas the number of abnormal images is less than 4000.

We used the finite impulse response bandpass filter (FIR) [52], a time-frequency transform plane, to transform a single-channel ECG signal into a four-channel ECG signal. Then, for each channel, we used sliding window segmentation on a sequence of segments of equal length to divide $x^{\left(i\right)}\varepsilon R^{n}$, as shown in Table 3.

### 3.2. Proposed Method

**Figure 9:**
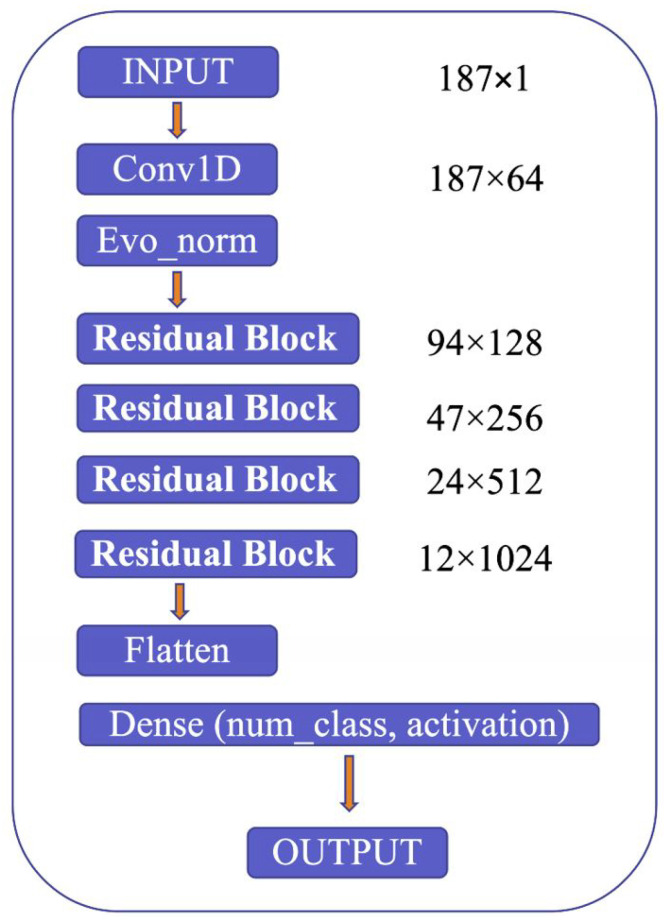
Model structure.

The proposed model’s architecture is detailed in Figure 9, which highlights several critical components, including the use of Convolution 1D (Conv1D) to expand the number of channels in the input image, evolving normalization–activation layers (Evo_norm) to normalize the output from the Conv1D block, and residual blocks to analyze the input features comprehensively. The model starts by resizing the input image to 187 × 1 and feeding it into the Conv1D block to expand the number of channels. The output from the Conv1D block is then passed through an Evo_norm block, followed by four residual blocks. The output from these blocks is then processed using the flatten and dense tools, which ultimately produce the final output.

Evo_norm has potential benefits, such as non-centered normalization, mixed variances, and tensor-to-tensor processing. It is a collection of normalization–activation layers combined into a single computation graph. Evo_norm is divided into two series: B (batch-dependent) and S (individual samples). These are explained in detail in a paper by Liu et al. [53].

**Figure 10:**
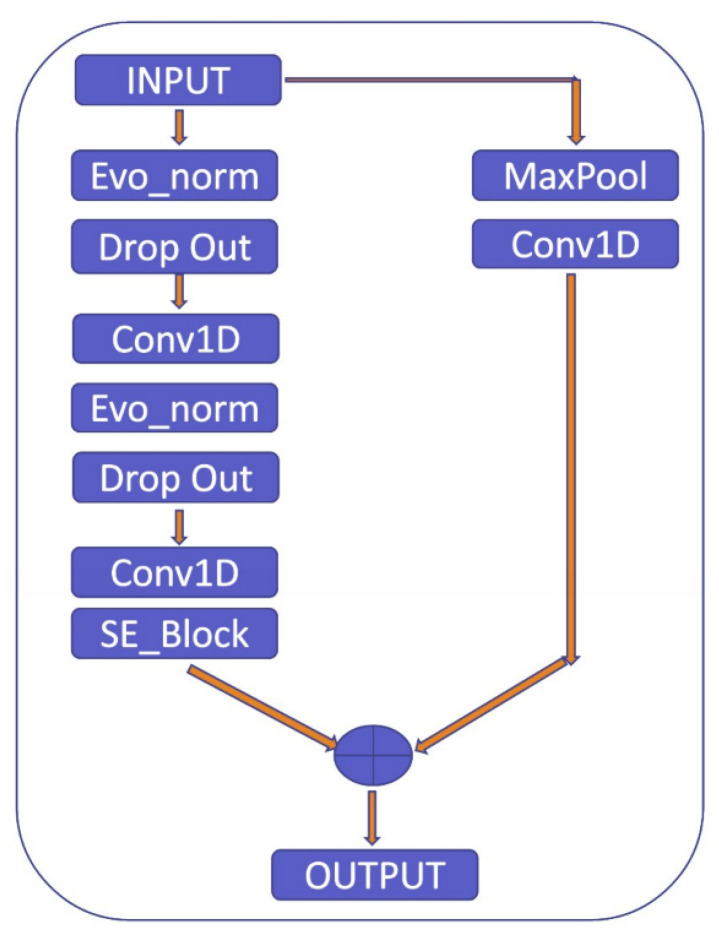
Residual block.

To clarify the explanation, the residual block used in the proposed model consists of two pathways: the first pathway involves the max pooling and Conv1D layers to extract features from the input, while the second pathway further refines these features using Evo_norm, Dropout, Conv1D, and SE_Block, as shown in Figure 10. The SE_Block is a squeeze-and-excitation block that adaptively recalibrates the channel-wise feature responses. The outputs from the two pathways are then multiplied element-wise to produce the final output of the residual block. By using this approach, the residual block is able to effectively capture and refine the important features in the input signal.

**Figure 11:**
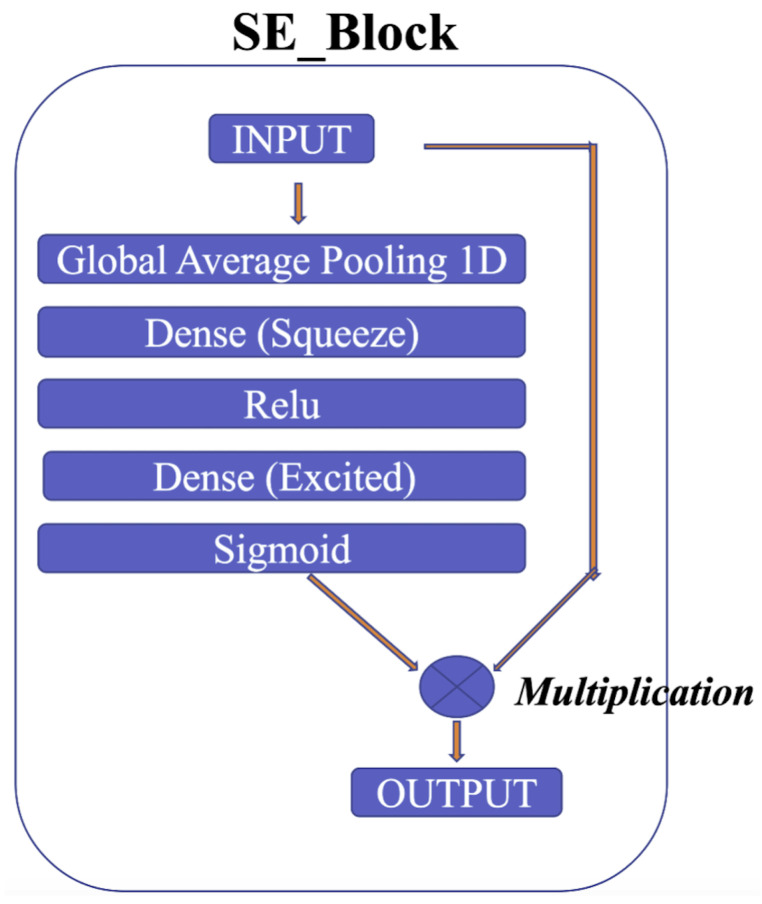
SE block.

The squeeze-and-excitation for Conv1D blocks (SE) [54] is one of the modules used to improve the model’s performance. The intricacies of the squeeze-and-excitation mechanism are shown in Figure 11. The main idea is that the squeeze operation is used to obtain a single value for each channel of input features, while the excitation operation on the output of the squeeze operation is used to obtain per-channel weights. In addition, this study proposes gradient clipping and a normalized gradient to enable faster convergence compared to traditional gradient descent with a fixed stepsize [55]. The experimental results for two popular ECG datasets demonstrate the benefits of the proposed model, which achieves an improved performance in distinguishing between different classes of ECG signals.

### 3.3. Evaluation Metrics

Following AAMI’s suggestion, we used accuracy, precision, and recall to evaluate the model’s efficiency.

(1) $$\mathrm{Accuracy}=\frac{\mathrm{TP}+\mathrm{TN}}{\mathrm{TP}+\mathrm{TN}+\mathrm{FP}+\mathrm{FN}}$$

(2) $$\mathrm{Precision}=\frac{\mathrm{TP}}{\mathrm{TP}+\mathrm{FP}}$$

(3) $$\mathrm{Recall}=\frac{\mathrm{TP}}{\mathrm{TP}+\mathrm{FN}}$$

True positive (TP) refers to an accurate identification of the positive outcome.

True negative (TN) refers to an accurate identification of the negative outcome.

False positive (FP) is a mistaken identification of the positive outcome.

False negative (FN) is a mistaken identification of the negative outcome.

### 3.4. Loss Function

For the PhysioNet MIT-BIH dataset, we applied the traditional loss, namely the categorical cross-entropy loss. For the PhysioNet PTB dataset, we used the binary focal loss. The categorical cross-entropy can be found through:

(4) $$CE=-log(\frac{e^{s_{p}}}{\sum_{j}^{C}e^{s_{j}}})$$

where $s_{p}$ is the positive class; $s_{j}$ is the score of the positive class; and C is the class.

The binary focal loss can be calculated as follows:

(5) $$FL\left(p_{t}\right)=-\propto_{t}{\left(1-p_{t}\right)}^{\propto }log\left(p_{t}\right)$$

where the class’s probability estimate from the model is given by p, and ∝ is a weighting factor.

### 3.5. Experiment Setup

#### 3.5.1. The PhysioNet MIT-BIH Dataset

For the PhysioNet MIT-BIH dataset, we perform training with five-fold cross-validation. We set up the experiment with the same details as for training one fold at a time. The chosen optimization technique is Adam. The initial value of the learning rate of 1 × 10^−3^ would be multiplied by 0.1 at the 20th and 40th epochs. The training runs for 50 cycles (or epochs).

#### 3.5.2. The PhysioNet PTB Dataset

We utilized 10-fold cross-validation for training on the PhysioNet PTB dataset. In each fold, we set up the experiment with the same details. The optimizer is the Adam method. The initial value of the learning rate of 1 × 10^−3^ would be multiplied by 0.1 at the 40th and 120th epochs. The number of epochs is 150.

#### 3.5.3. The PhysioNet Challenge 2017 Dataset

This dataset was divided into three sets: a training set (75%), a validation set (10%), and a test set (15%), which were used to train and evaluate the model. To address the class imbalance, we converted each ECG recording into sliding frames of the length 3000, using 50 and 500 steps for recordings from AF and non-AF ECGs, respectively. The model was trained for 25 epochs with a batch size of 64 and a learning rate of 0.001, with an early stopping decay of 0.9. We used ADAM optimization and binary cross-entropy as the loss function. To evaluate the model, we tested it five times and report the mean values with one standard deviation.

#### 3.5.4. Hyperparameters

The model’s hyperparameters were chosen based on the suggestions from the reference papers. The SE ratio was set to 0.25, and the hyperparameters ∝ = 2 and $\propto_{t}$ = 0.25 were used for the focal loss. During training, we applied oversampling to both the training and validation data to address the class imbalance. Additionally, we trimmed the gradient based on its norm with a threshold of 0.001. For the test data, which were independent or unseen, we analyzed the results and found them to be promising.

#### 3.5.5. Independent Testing Set

**Figure 12:**
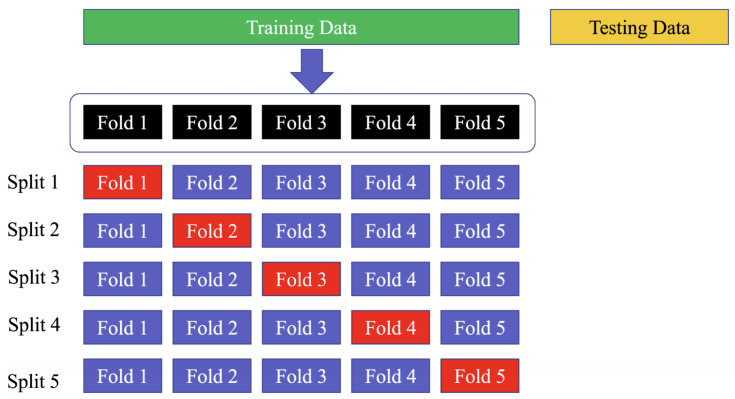
Divide the dataset into training, testing, and validation sets. The blue folds are the training set, while the red fold is the validation set.

Using a five-fold cross-validation technique on the training set means that the data are divided into five equal parts or “folds”, and the model is trained and validated five times, with a different fold being used for validation each time. This allows for a more comprehensive evaluation of the model’s performance on the data, as each data point is used for validation at least once. Similarly, employing a 10-fold cross-validation strategy on the PhysioNet MIT-BIH dataset means that the data are divided into ten equal parts, and the model is trained and validated ten times, with a different fold being used for validation each time. This strategy can help to ensure that the model’s performance is not affected by the particular set of data used for training and validation, as each data point is used for validation at least once across all folds. The use of the “sklearn.model selection.StratifiedKFold” function ensures that the class distribution is preserved in each fold of the dataset, which is important for ensuring a fair evaluation of the model’s performance. Figure 12 shows the way to divide the data in the training, testing, and validation sets.

## 4. Results

The experimental results are examined on two ECG datasets, namely, the PhysioNet MIT-BIH Arrhythmia and PhysioNet PTB Myocardial Infarction.

### 4.1. The Contribution of Evolving Normalization–Activation (EVO), Squeeze-and-Excitation (SE), and Gradient Clipping (GC)

Table 4 shows the contribution of each component to the performance of the proposed method. The results indicate that the Evo_norm block plays a significant role in enhancing the classification accuracy of the three datasets. The addition of the SE block also improves the performance on these three datasets. Finally, gradient clipping also shows a positive effect on the performance of the model. Overall, the combination of all three components results in the best performance on the three datasets.

It can be seen that the proposed model with all three components achieved the highest performance with an accuracy of 98.56%, followed by the model without GC with an accuracy of 98.42%, the model without SE with an accuracy of 98.36%, and the model without EVO with an accuracy of 95.50%. This indicates that each component has a contribution to the performance of the model, with EVO having a higher impact than SE and GC. The combination of all three components significantly improved the efficiency of the model in this study.

### 4.2. The PhysioNet MIT-BIH Arrhythmia Classification

#### 4.2.1. Comparative Results

In an effort to facilitate comparison, our study was benchmarked against the work presented in [51]. The results of our study revealed an average accuracy of 98.5%, which is significantly higher than the accuracy scores obtained in previous studies, such as [51,56,57], as shown in Table 5. These results are strong evidence that our research has an advanced benefit for arrhythmia classification.

#### 4.2.2. Confusion Matrix

**Figure 13:**
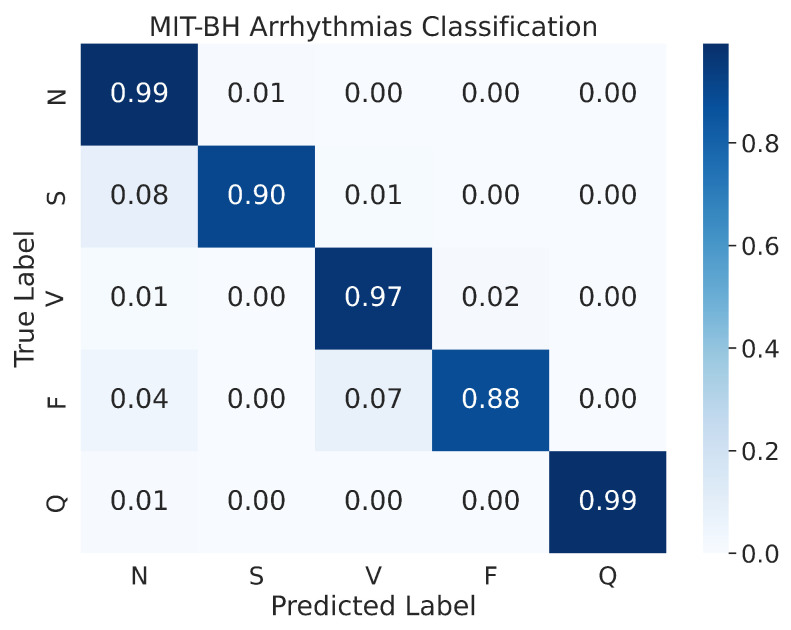
The confusion matrices for MIT-BH Arrhythmias classification.

In a prior study by Kachuee et al. [51], the authors presented the confusion matrix of the resampled testing data. Our study, however, uses the actual testing set to evaluate the performance of the model, and the results indicate an improvement in performance. As shown in Figure 13, the confusion matrix of the MIT-BIH Arrhythmia classification for the five heartbeat classes (N, S, V, F, and Q) is presented. Despite evaluating the performance on the actual testing set, instead of just the resampled testing data as in previous studies, our proposed model still demonstrates superior performance. The model demonstrates an accuracy of 99% for the N and Q classes, 97% for the V class, 90% for the S class, and 88% for the F class. As mentioned above, the MIT-BIH Arrhythmia dataset exhibits a class imbalance, yet our proposed model achieved high accuracy in both scenarios where there were ample input images (i.e., class N) or limited input images (i.e., class Q). This suggests that our proposed model is effective in classifying ECG signals from different heartbeat classes.

### 4.3. The PhysioNet PTB Myocardial Infarction Classification

#### 4.3.1. Comparative Result

In this study, we conducted an empirical comparison of our proposed model against other previous studies, as presented in Table 6. The evaluation of the results was conducted using three common performance metrics, namely accuracy, precision, and recall. The results showed that our proposed model exhibited a superior classification performance, achieving an accuracy of 98.28. Furthermore, our model performed exceptionally well in terms of recall, with a score of 97.72, which was the highest among all the studies compared. One of the most notable achievements of our model was its precision, which was measured at 99.90, meaning that the model only made 0.01% of mistaken classifications, making it the best-performing model among the previous studies.

#### 4.3.2. Confusion Matrix

**Figure 14:**
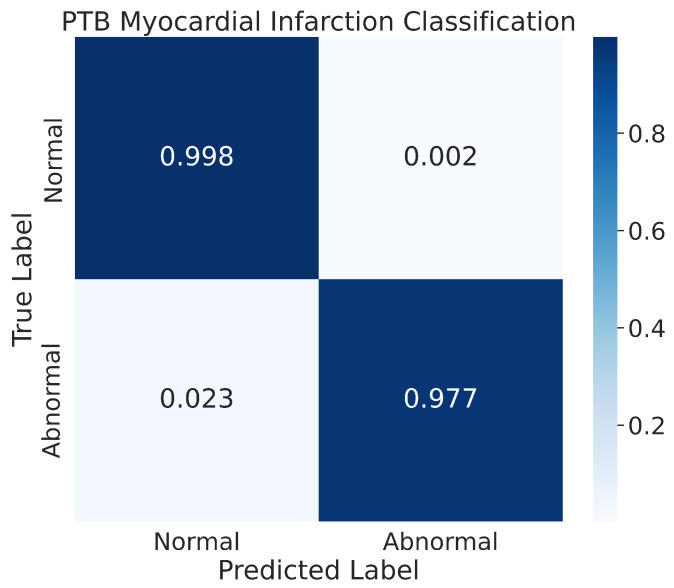
The confusion matrix for the PhysioNet PTB Myocardial Infarction classification.

The results of our model’s classification performance on the PhysioNet PTB Myocardial Infarction data are depicted in Figure 14. The figure represents the confusion matrix for the two classes, normal and abnormal, that were evaluated. The results indicate that our model achieved high accuracy in classifying the normal class, with 99.8% of the normal images being correctly classified and only a small percentage, 0.2%, being misclassified. Additionally, our model also showed good performance in recognizing the abnormal class, with 97.7% of the images in that class being correctly classified. The remaining images in the abnormal class were misclassified by the model during the prediction process.

### 4.4. Atrial Fibrillation (AF) Anomaly Detection in PhysioNet Challenge 2017 Dataset

Table 7 compares the performance of our proposed model with the existing models in the literature. Our model achieved an F1-score of approximately 86.71%, outperforming other models, such as MINA, CRNN, and EXpertRF, which achieved F1-scores of 83.42%, 82.62%, and 81.80%, respectively. These results demonstrate the superiority of our proposed model over previous studies.

## 5. Conclusions

In this study, we present a novel approach for ECG heartbeat classification. Our proposed model outperforms the existing state-of-the-art techniques, achieving superior accuracy results on both the PhysioNet MIT-BIH and PTB datasets. The high performance of our model is attributed to the combination of the Convolution 1D (Conv1D), evolving normalization–activation layers (Evo_norm), and the residual block module, with accuracy rates of 98.5% and 98.28%, respectively, on these datasets. Furthermore, we evaluated the model’s efficiency on the PhysioNet Challenge 2017 dataset and achieved an F1-score of approximately 86.71%, outperforming the existing models. Overall, our proposed model is a valuable contribution to ECG heartbeat classification.

In future work, we aim to evaluate the effectiveness of our model on additional datasets and explore optimizing the model’s architecture with fewer parameters. We believe that our model has the potential to be useful not only for ECG heartbeat classification but also for general classification tasks in wearable device applications.

## Figures and Tables

**Table 1:** Mappings between beat annotations and AAMI EC57 [51] categories.

Category	Annotations
N	Normal Left/Right bundle branch block Atrial escape Nodal escape
S	Atrial premature Aberrant atrial premature Nodal premature Supra-ventricular premature
V	Premature ventricular contraction Ventricular escape
F	Fusion of ventricular and normal
Q	Paced Fusion of paced and normal Unclassifiable

**Table 2:** Number of recordings for AF patients and non-AF candidates.

Type	Training	Validation	Testing
AF	564	70	124
Non-AF	5832	782	1156

**Table 3:** Number of segments for AF patients and non-AF candidates.

Type	Training	Validation	Testing
AF-segments	76,585	8069	17,044
Non-AF-segments	79,080	11,039	15,631

**Table 4:** Performance comparison on PhysioNet MIT-BIH with three components.

	Proposed Model	without EVO	without SE	without GC
Average Accuracy	98.56%	95.50%	98.36%	98.42%

**Table 5:** Comparative results in the PhysioNet MIT-BIH Arrhythmia classification.

Work	Average Accuracy (%)
Kachuee et al. [51]	93.4
Acharya et al. [56]	93.5
Martis et al. [57]	93.8
Li ei al. [58]	94.6
Ganguly et al. [47]	97.3
This paper	98.5

**Table 6:** Comparative results in the PhysioNet PTB Myocardial Infarction classification.

Work	Accuracy (%)	Precision (%)	Recall (%)
Kachuee et al. [51]	95.9	95.2	95.1
Acharya et al. [59]	93.5	92.8	93.7
Safdarian et al. [60]	94.7	-	-
Kojuri et al. [61]	95.6	97.9	93.3
Sun et al. [62]	-	82.4	92.6
Liu et al. [63]	94.4	-	-
Sharma et al. [64]	96	99	93
This paper	98.28	99.90	97.72

**Table 7:** Comparative results for atrial fibrillation (AF) anomaly detection.

Method	F1-Score
ExpertRF [65]	81.80 ± 0.0
CRNN [65]	82.62 ± 0.0215
MINA [65]	83.42 ± 0.0229
Ours	86.71 ± 0.985

## Data Availability

The data presented in this study are openly available at https://www.physionet.org/content/mitdb/1.0.0/ [49] accessed on 24 February 2005, at https://www.physionet.org/content/ptbdb/1.0.0/ [50] accessed on 25 September 2004, and at https://physionet.org/content/challenge-2017/1.0.0/ [25] accessed on 1 February 2017.
